# Blunt Cardiac Injury in Trauma Patients with Thoracic Aortic Injury

**DOI:** 10.1155/2011/848013

**Published:** 2011-07-14

**Authors:** Rathachai Kaewlai, Marc A. de Moya, Antonio Santos, Ashwin V. Asrani, Laura L. Avery, Robert A. Novelline

**Affiliations:** ^1^Department of Radiology, Massachusetts General Hospital, 55 Fruit Street, FND-210, Boston, MA 02114, USA; ^2^Department of Radiology, Ramathibodi Hospital and Mahidol University, 270 Rama VI Road, Ratchatewi, Bangkok 10400, Thailand; ^3^Department of Surgery, Massachusetts General Hospital, 55 Fruit Street, FND-210, Boston, MA 02114, USA; ^4^Departamento de Cirurgia Geral, Hospital Geral de Santo António, Largo do Prof. Abel Salazar, 4099-001 Porto, Portugal

## Abstract

Trauma patients with thoracic aortic injury (TAI) suffer blunt cardiac injury (BCI) at variable frequencies. This investigation aimed to determine the frequency of BCI in trauma patients with TAI and compare with those without TAI. All trauma patients with TAI who had admission electrocardiography (ECG) and serum creatine kinase-MB (CK-MB) from January 1999 to May 2009 were included as a study group at a level I trauma center. BCI was diagnosed if there was a positive ECG with either an elevated CK-MB or abnormal echocardiography. There were 26 patients (19 men, mean age 45.1 years, mean ISS 34.4) in the study group; 20 had evidence of BCI. Of 52 patients in the control group (38 men, mean age 46.9 years, mean ISS 38.7), eighteen had evidence of BCI. There was a significantly higher rate of BCI in trauma patients with TAI versus those without TAI (77% versus 35%, *P* < 0.001).

## 1. Introduction

Blunt cardiac injury (BCI) is a very rare, but potentially fatal, condition that accounts for 12%–32% of trauma-related fatality [1–3]. Ruptured cardiac cavities, coronary arteries, or intrapericardial portion of major vessels typically result in death at the scene of the collision [2, 4]. Victims of relatively less severe cardiac injuries such as myocardial contusion, hemopericardium due to contusions or lacerations, valvular regurgitation, or myocardial infarction due to coronary artery injury may survive the initial trauma and thus present to the emergency department (ED) for evaluation [1, 5, 6]. Among those who arrived alive at the ED, BCI is associated with a high mortality rate of 89% [7]. The frequency of reported BCI in trauma patients is difficult to determine. It varies widely from 0.045% to 86%, depending on patient population, subpopulation, and a variety of factors that were considered to make the diagnosis [1, 4, 5, 7–10]. Patients who suffer BCI usually have multiple severe concomitant injuries of other organs, including the thoracic aorta. By itself, thoracic aortic injury (TAI) is considered a potentially fatal condition if left untreated [1, 11–15]. Therefore, a surgical or endovascular intervention is usually performed as a definitive treatment if the patient's condition allows. When an open thoracotomy or endovascular repair is expected, it is critical for treating physicians to evaluate all associated injuries to determine the need for immediate interventions and for preoperative risk assessment [16]. With the widespread use of CT in polytrauma patients, the diagnosis of TAI and coexisting injuries to the lungs, pleura, chest wall, and abdomen can quickly and accurately be made on a single scan [13, 17]. However, concurrent injuries to the heart in patients with TAI may go undetected on clinical examinations and on imaging due to the subtlety of the injuries. Existing data suggests a wide range of incidence of BCI in trauma patients with TAI between 3.6% and 63% [11, 14, 16]. By using diagnostic criteria of BCI based on abnormal ECG, elevated Creatine Kinase-MB (CK-MB), and/or abnormal echocardiography, we performed a retrospective investigation of trauma patients with TAI to (1) determine the frequency of BCI in trauma patients with TAI; (2) assess whether BCI was significantly more prevalent among trauma patients with TAI than those without TAI in whom the abbreviated injury scales (AIS) were comparable.

## 2. Materials and Methods

### 2.1. Subjects

Our hospital institutional review board approved the investigation, which was deemed HIPAA compliant. Patients with a diagnosis of TAI from January 1999 to May 2009 were identified from the hospital's trauma registry. Patients with available ECG and serum Creatine Kinase-MB (CK-MB) performed within 24 hours of arrival at the hospital were included in the investigation. TAI was either diagnosed preoperatively with contrast-enhanced computed tomography (CT) of the thorax, with angiography, or at surgery. The diagnosis of TAI was made on CT scan when there was aortic pseudoaneurysm and/or intimal flap involving the thoracic aorta. All patients with TAI, diagnosed on CT were confirmed at the time of therapeutic angiography or surgery. 

Patient's age, gender, history of cardiac disease, mechanism of trauma, abbreviated injury scales (AIS), injury severity scores (ISS), treatment of TAI and outcome including survival at 24 hours and at 30 days of arrival to the hospital were acquired from electronic medical records. Results of electrocardiography (ECG) performed within 24 hours of arrival, cardiac enzyme assays performed within 72 hours of arrival, and echocardiography performed within one week of arrival were collected for evidence of BCI. Patients with TAI were compared with a control population who did not have TAI. A list of potential control population was acquired by a search of the trauma registry for consecutive trauma patients from January 2006 to April 2009. Based on this list, we selected two control cases per one TAI patient by matching the abbreviated injury severity (AIS) for head (±1), chest (±1), extremity (±1), face (±2), abdomen (±2), and external (±1) to include in the control group. 

The diagnosis of blunt cardiac injury in both case and control populations was based on ECG, serum CK-MB, and/or echocardiography. BCI was considered present if there was an ECG abnormality with either CK-MB elevation or abnormal echocardiography. ECG abnormality was considered a necessary component of diagnosing BCI. *Abnormal admission ECG* was defined as presence of nonspecific ST-T changes, premature atrial contractions, premature ventricular contractions, heart block, or ischemic changes in trauma patients without underlying heart disease. In patients with underlying heart disease, these changes were considered positive when they were not present on prior ECGs. *Abnormal CK-MB level* was found to correlate directly with cardiac complications from blunt cardiac trauma requiring treatment, therefore it was used as a criterion in this investigation. Abnormal CK-MB was defined as an elevated level above normal (0.0–6.9 ng/mL) [18]. Some patients underwent serial CK-MB assay, in these patients we recorded only the peak CK-MB within the first 24 hours of admission. *Abnormal echocardiography* was defined as at least mild degree of valvular insufficiency, presence of thrombus, wall hypokinesia, pericardial effusion, and septal rupture [19, 20]. If there was a prior echocardiography, changes were considered abnormal if it was new. When present, BCI was graded according to the American Association for the Surgery of Trauma Organ Injury Scale (OIS) [21], which is based on ECG and pathology. Because none of our patients had autopsy, we utilized data from echocardiography as a pathological criteria. The classification is summarized in Table 1. 

### 2.2. Statistical Calculation

Mean, standard deviation, and range were calculated for continuous variables, using Microsoft Excel (Microsoft Corp., Redmond, Wash, USA). Frequencies and relative frequencies were calculated for noncontinuous variables. Differences of categorical variables and continuous variables were tested by Fisher's exact and student *t*-tests, respectively, using QuickCalcs (GraphPad Software Inc., La Jolla, Calif, USA).

## 3. Results

From January 1999 to May 2009, there were 39 patients diagnosed with TAI. Twenty-five cases were diagnosed on CT and confirmed at endovascular treatment or surgery. The remaining were diagnosed with angiography (7 cases) and surgery (7 cases). Of these, 26 patients (19 men, 7 women, mean age 45.1 years, SD 21.2, range 20–98 years) had available admission ECG and serum CK-MB; therefore they were included in the investigation as the *study group*. The mean AIS for the head, chest, extremity, face, abdomen, and external were as follows: 1.62, 4.31, 2.04, 0.5, 1.62, and 0.73, respectively. The mean injury severity score (ISS) was 34.4 (SD 11.1, range 17–57).

There were 52 control cases (38 men, 14 women, mean age 46.9 years, SD 15.7, range 17–81). The mean AIS for the head, chest, extremity, face, abdomen, and external were as follows: 1.81, 4.17, 1.83, 0.37, 1.29, and 0.67, respectively. The mean injury severity score (ISS) was 32.6 (SD 11.1, range 16–59).

The detailed characteristics of study and control groups are provided in Table 2. There was no significant difference in mean age, gender, presence of underlying cardiac disease, mean AIS, and mean ISS between the two groups. Patients in the study group had a higher rate of trauma from motor vehicle collision and a longer mean length of stay than those in the control population. The abnormalities found on ECG, cardiac enzyme assay, and echocardiography are provided in Table 3. Frequency of ECG abnormalities in the study group was not statistically different from the control groups (80.8% versus 73.1%, *P* = 0.787). However, there was a higher rate of abnormal CK-MB in the study group (24/25, 96%) than in the control group (27/52, 51.9%, *P* < 0.001). Echocardiography was utilized in 15 study patients and 10 control patients. They were considered abnormal in three cases of the study group and two of the control group (20%). Among these five cases, echocardiography contributed to one additional case of BCI in the study group and none in the control group. Using criteria defined above, blunt cardiac injury was diagnosed in 20 patients with TAI (77%) and 18 patients without TAI (35%) with a *P* value of less than <0.001. Of note, the death rate in patients with combined TAI and BCI did not significantly differ from those with BCI alone (7.7% versus 3.8%, *P* = 0.606).

Analysis of patients who were excluded from the study group (*n* = 13) showed that they were predominantly male (*n* = 9), had a mean age of 45.2 years (SD 20.5, range 17–92), and with mean AIS scores for the head, chest, extremity, face, abdomen, and external of 1.62, 4.62, 2.31, 0.23, 1.31 and 0.38, respectively. Six patients had serum CK-MB performed, three had echocardiography, and none had ECG. All CK-MB levels and echocardiography were abnormal. For the treatment of TAI, 4 patients underwent surgery, 6 had endovascular therapy, and three received supportive measures only. All patients who had endovascular therapy survived but the remaining patients deceased within their first 24 hours of admission. Autopsy was not performed in any of the deceased patients.

## 4. Discussion

Blunt cardiac injury is a potentially lethal injury that may occur in trauma patients with thoracic aortic injury. A high clinical suspicion of BCI in this patient subset is important to achieve the diagnosis [10, 19, 22, 23]. The incidence of combined TAI and BCI varies widely from 4% to 61.5% [11, 14, 16] depending on diagnostic criteria. Fabian et al. [11] reported 4% prevalence of BCI in 274 patients with TAI. Cook et al. [14] showed 18%–26% frequency of BCI in patients with TAI, diagnosed with echocardiography, serum troponin I, operative description, need for inotropic support, and ongoing treatment for angina pectoris to conclude the presence of cardiac injury. Kram et al. [16] described eight (out of 13) trauma patients with TAI who had concomitant BCI diagnosed by ECG, cardiac enzyme assay, and radionuclide ventriculography. Our investigation confirmed, by utilizing a case-control study, that patients with TAI were significantly more likely to have concomitant blunt cardiac injuries with a frequency of 77%, compared with 35% in those without TAI who had comparable degree of traumatic injuries.

Our investigation has several limitations. The criteria used to define blunt cardiac injury have often been challenged because neither ECG nor cardiac enzymes are specific for cardiac injury in blunt trauma [22, 24–27]. Albeit, in clinical practice, they are commonly performed as it was evident in our investigation. Serum troponin I, believed to be highly specific for cardiac injury [28–30], was not consistently obtained in our patients during the investigation period. Therefore, we opted to use such criteria for diagnosis of blunt cardiac injury in our cases. We excluded approximately one third of patients with TAI from the analysis because they did not undergo ECG and serum CK-MB at the time of admission. The reasons excluded patients did not receive these diagnostic tests were unclear but it might be due to factors such as variation of care from one to another physician, acuity of patient's presentation eluding the ability to perform these tests before operative interventions, or a lack of standardized protocol to assess for BCI in our institution at the time of investigation. More patients in the study group had echocardiography performed compared with the control group (15 of 26 in the study group, 10 of 52 in the control group), which may introduce detection bias into the investigation. Given a small sample size and retrospective nature, we cannot conclude that the presence of BCI in TAI patients would alter clinical management or patient outcome. In our patient population, the majority of patients with TAI had concomitant BCI; therefore, there was not enough number of patients with isolated TAI to perform a meaningful comparison. The presence of BCI in patients with TAI, however, had been described in the literature as associated with high morbidity and mortality [16], high rate of hypotension [31], ICU admission [32], and increased risk of cardiac or operative intervention [18]. Although our data suggested considering an outcome as short-term survival, death rates of patients with combined TAI and BCI were not different from those with BCI alone. We were unable to differentiate BCIs that were clinically significant from those that were not based on our present data. The diagnosis of BCI was based on clinical tests, not pathological evaluation of the heart. Issues remain for the management of those who had blunt cardiac injuries, whether the knowledge of BCI changes the management plan or patient outcome. Further investigation with prospective design and a larger patient cohort would be required to answer these important questions.

In conclusion, blunt cardiac injury (diagnosed by a combination of ECG, serum CK-MB, and/or echocardiography) was significantly more frequent in patients with thoracic aortic injury than in patients without thoracic aortic injury.

## Figures and Tables

**Table 1:** Grading of cardiac injury by the American Association for Surgery of Trauma. Adapted from [21].

Grade	ECG	Pathology
I	Nonspecific ST or T wave change, premature atrial or ventricular contraction, persistent sinus tachycardia	Blunt or penetrating pericardial wound without cardiac injury, tamponade, or herniation

II	Heart block, ischemic changes without cardiac failure	Penetrating tangential cardiac wound up to but not extending through endocardium without tamponade

III	Sustained or multifocal ventricular contractions	

IV		Blunt or penetrating cardiac injury with septal rupture, pulmonary or tricuspid incompetence, papillary muscle dysfunction, or distal coronary artery occlusion producing cardiac failure
		Blunt or penetrating cardiac injury with aortic or mitral incompetence
		Blunt or penetrating cardiac injury of the right ventricle, right or left atrium

V		Blunt or penetrating cardiac injury with proximal coronary artery occlusion
		Blunt or penetrating left ventricular perforation
		Stellate injuries <50% tissue loss of the right ventricle, right or left atrium

VI		Blunt avulsion of the heart

**Table 2:** Comparison between patients with TAI (study group) and without TAI (control group).

	Study group (*n* = 26)	Control group (*n* = 52)	*P* value
Mean age (years) (SD, range)	45.1 (21.2, 20–98)	46.9 (15.7, 17–81)	0.673
Male gender	19	38	1.000
Mechanism of trauma			0.001
Motor vehicle collision	23	26	
Fall	0	18	
Others	3	8	
Presence of underlying cardiac disease	4	2	0.091
Mean length of stay (days) (mean, SD, range)	25.1 (21.9, 0–84)	12.5 (10.9, 0–43)	0.001
Mean abbreviated injury scales (SD, range)			
Head	1.62 (1.9, 0–5)	1.81 (2.0, 0–5)	0.689
Chest	4.31 (0.5, 4-5)	4.17 (0.5, 3–5)	0.247
Extremity	2.04 (1.2, 0–3)	1.83 (1.2, 0–4)	0.469
Face	0.50 (0.8, 0–2)	0.37 (0.7, 0–2)	0.463
Abdomen	1.62 (1.6, 0–5)	1.29 (1.5, 0–4)	0.373
External	0.73 (0.5, 0–2)	0.67 (0.5, 0–2)	0.619
Mean injury severity score (SD, range)	34.42 (11.1, 17–57)	32.60 (11.1, 16–59)	0.497

Treatment of TAI		N/A	
Surgery	12		
Endovascular	12		
Medical	2		

Outcome			0.326
Survive	23	50	
Died within 24 hours of arrival	2	2	
Died within one month	1	0	

**Table 3:** Details of cardiac test results including ECG, CK-MB assay, and echocardiography.

	Study group (*n* = 26)	Control group (*n* = 52)	*P* value
Number with ECG abnormalities	21 (80.8%)	38 (73.1%)	0.787
Type of ECG abnormalities*			
Nonspecific ST-T changes	17	33	
Heart block	8	10	
Ischemic changes	2	1	
Atrial arrhythmias	2	7	

Number with abnormal CK-MB level	24 (24/25, 96%)**	27 (51.9%)	<0.001
Mean CK-MB level when elevated (ng/mL) (SD, range)	95.2 (309, 8.6–1544)	25.8 (23.5, 7.2–109.3)	0.109

Number with abnormal echocardiography***	3 (3/15, 20%)	2 (2/10, 20%)	1.000
Type of echocardiographic abnormalities			
Valvular insufficiencies	3	1	
Pericardial effusion	0	1	
Wall motion abnormalities	0	1	

Number with blunt cardiac injury	20 (20/26, 77%)	18 (18/52, 35%)	<0.001

*Numbers are not mutually exclusive. **CK-MB level was not tested in one subject, in whom an echocardiography was performed. ***Echocardiography was performed in 15 and 10 patients in the study and control groups, respectively.
